# CCN1 Promotes Inflammation by Inducing IL-6 Production *via* α6β1/PI3K/Akt/NF-κB Pathway in Autoimmune Hepatitis

**DOI:** 10.3389/fimmu.2022.810671

**Published:** 2022-04-25

**Authors:** Renquan Jiang, Jifeng Tang, Xuehao Zhang, Yujue He, Ziqing Yu, Shuhui Chen, Jinfang Xia, Jinpiao Lin, Qishui Ou

**Affiliations:** ^1^ Department of Laboratory Medicine, Gene Diagnosis Research Center, The First Affiliated Hospital, Fujian Medical University, Fuzhou, China; ^2^ Fujian Key Laboratory of Laboratory Medicine, The First Affiliated Hospital, Fujian Medical University, Fuzhou, China

**Keywords:** AIH (autoimmune hepatitis), CCN1 (cellular communication network factor 1), IL-6 (interleukin 6), inflammation, NF-kappa B

## Abstract

Autoimmune hepatitis (AIH) is a chronic inflammatory liver disease with unknown etiology. CCN1, an extracellular matrix-associated protein, is associated with carcinoma, inflammation, liver fibrosis, and even autoimmune diseases. However, the role that CCN1 plays in AIH has remained undetermined. In this study, expression of CCN1 in liver was detected by real-time PCR, western blot and immunohistochemistry (IHC). CCN1 level in serum was detected by ELISA. Diagnostic value of CCN1 was determined by receiver operating characteristic (ROC) curve analysis. CCN1 conditional knockout (CCN1*^fl/fl^* Cre^+^) mice were generated by mating CCN1*^fl/fl^* C57BL/6J and CAG-Cre-ERT C57BL/6J mice. Autoimmune hepatitis mice model was induced by concanavalin A (ConA). IKKα/β, IκBα, NF-κB p65 and Akt phosphorylation were determined by western blot. NF-κB p65 nuclear translocation was examined by immunofluorescence. Here, we found that CCN1 was over-expressed in hepatocytes of AIH patients. CCN1 level also increased in serum of AIH patients compared to healthy controls (HC). ROC curve analysis results showed that serum CCN1 was able to distinguish AIH patients from HD. In ConA induced hepatitis mice model, CCN1 conditional knockout (CCN1*^fl/fl^* Cre^+^) attenuated inflammation by reducing ALT/AST level and IL-6 expression. *In vitro*, CCN1 treatment dramatically induced IL-6 production in LO2 cells. Moreover, the production of IL-6 was attenuated by CCN1 knockdown. Furthermore, we showed that CCN1 could activate IL-6 production *via* the PI3K/Akt/NF-κB signaling pathway by binding to α6β1 receptor. In summary, our results reveal a novel role of CCN1 in promoting inflammation by upregulation of IL-6 production in AIH. Our study also suggests that targeting of CCN1 may represent a novel strategy in AIH treatment.

## Introduction

Autoimmune hepatitis (AIH) is an inflammatory liver disease characterised by interface hepatitis and autoantibody positivity (1). The etiology of autoimmune hepatitis is complicated and unclear, but genetic, epigenetic and environmental factors may be implicated in its pathogenesis (2–4). It is now widely known that the liver injury of AIH is induced by an immune tolerance breakdown and autoimmune response against hepatocytes. The initiation of the self-attack and the dysregulation of the immune system in the liver microenvironment play roles in the process of liver damage (3, 5). T helper (Th) cells have an important role in triggering the self-attack process by recognizing the autoantigens, and then B cells are responsible for the subsequent production of autoantibodies (6). It has been reported that an imbalance between Treg and effector T cells is related to AIH pathogenesis (7, 8). Moreover, the Treg/Th17 ratio has been regarded as a predictor of the degree of liver inflammation, as well as a therapeutic target in AIH (9). In recent years, it has also been widely studied that cells and cytokines involved in immunoregulation maintain the liver immunologic balance to protect the liver from inflammatory damage (6, 10). Proinflammatory cytokines such as interferon(IFN)-γ, interleukin(IL)-12, tumor necrosis factor(TNF)-α, IL-6 and IL-23 have been implicated in the pathogenesis of autoimmune hepatitis (11). These reports suggest that imbalance of immune cells and cytokines plays an important role in AIH. However, further studies are required to elucidate their exact roles in the AIH disease process.

Given the complexity of the disease, the difficulty of confirmatory diagnosis and the lack of valid and/or the long development times of current animal models for AIH research (12, 13), the related basic and clinical researches are difficult to carry out. Furthermore, there is no effective therapy for AIH patients except for standard immunosuppressive treatment using corticosteroids with or without azathioprine (14, 15). However, not all patients respond well to this treatment, and some patients will develop disease relapse after drug withdrawal (16). Recently, iimmunoregulatory therapy has also been explored as a potential therapy for AIH (17, 18). Intervention targeting immunomodulatory molecules may provide new ideas for the treatment of AIH.

CCN1 is a secreted, heparin-binding protein which is involved in a variety of diseases, including carcinoma (19), bacterial clearance (20) and inflammation (21). As a secreted extracellular matrix protein, CCN1 could play diverse roles by binding to different types of integrin molecules such as αvβ3, αvβ5, α6β1 and toll-like receptor (TLR) 2/4 on different cells (20, 22–24). We previously demonstrated that the overexpression of CCN1 stimulated by IL-17 promotes fibroblast-like synoviocytes (FLS) proliferation, and CCN1 can also stimulate FLS to produce IL-6, in turn, promote Th17 cell differentiation (24, 25). Moreover, p53 is involved in the posttranscriptional regulation of CCN1 expression *via* miRNA-22 (26). Studies show CCN1 has also significant effects on hepatic pathogenesis, such as biliary repair (27), liver fibrosis and inflammation (10, 28). These studies suggest that CCN1 may play an immunomodulatory role in immune diseases. Nevertheless, whether CCN1 has any effect on the inflammation process of AIH remains unknown. In this study, we aimed to explore the role of CCN1 in AIH. We will first examine the expression and diagnostic value of CCN1 in AIH patients, further verify the role of CCN1 in AIH through *in vivo* and *in vitro* experiments, and explore its possible signal pathways, so as to provide new ideas for the diagnosis and treatment of AIH.

## Materials and Methods

### Patients and Specimens

A total of 59 AIH patients, 26 primary biliary cholangitis (PBC) patients, 22 overlap syndrome (OS) patients and 68 healthy controls (HC) were included in the study. The diagnosis of AIH fulfilled the diagnostic criteria and index by Chinese Society of Hepatology, Chinese Society of Gastroenterology and Chinese Society of Infectious Diseases. Correspondingly, the diagnosis of PBC and OS fulfilled their classification criteria (29). All participants who had history of cardiovascular disease, endocrine disease, non-alcoholic steatohepatitis and alcoholic steatohepatitis, renal disease and any other chronic inflammatory diseases were excluded. Autoantibody characteristics of the selected participants were indicated in **Table 1**. The study was approved by the Institutional Medical Ethics Review Board of the First Affiliated Hospital of Fujian Medical University, Fuzhou, China (MTCA, ECFAH of FMU (30) 084-1). All participants provided written informed consent and identified by number.

**Table 1:** Autoantibody characteristics of AIH patients, PBC patients and OS patients.

	AIH	PBC	OS	P^1^	P^2^
Sex (M/F)	10/49	3/23	1/21	0.746	0.273
Age (years), median (IQR)	54 (16.5)	52.5 (12.25)	55 (22.25)	0.731	0.421
anti-SMA, %	13/54 (24.1%)	0/19 (0%)	0/16 (0%)	0.063	0.114
ANA, %	33/54 (61.1%)	14/19 (73.7%)	10/16 (62.5%)	0.139	1.000
anti-Ro52, %	13/48 (27.1%)	8/23 (34.8%)	11/16 (68.8%)	0.618	<0.05
anti-SLA, %	1/48 (2.1%)	0/23 (0%)	1/16 (6.3%)	1.000	0.440
anti-PML, %	2/48 (4.2%)	1/23 (4.3%)	5/16 (31.3%)	1.000	<0.05
anti-gp210, %	3/48 (6.3%)	10/23 (43.5%)	8/16 (50%)	<0.05	<0.05
anti-sp100, %	5/48 (8.3%)	3/23 (13.0%)	3/16 (18.8%)	0.585	0.374
AMA-M2-3E, %	5/48 (8.3%)	17/23 (73.9%)	9/16 (56.3%)	<0.05	<0.05
AMA-M2, %	4/48 (8.3%)	15/23 (65.2%)	6/16 (37.5%)	<0.05	<0.05
anti-LC-1, %	2/48 (4.2%)	0/23 (0%)	2/16 (12.5%)	1.000	0.258
anti-LKM-1, %	0/48 (0%)	0/23 (0%)	0/16 (0%)	/	/

AIH, autoimmune hepatitis; PBC, primary biliary cholangitis; OS, overlap syndrome.

P^1^, the difference between AIH and PBC group; P^2^, the difference between AIH and OS group.

### Animals

SPF-grade CCN1*^flox/flox^* Cre^+^ homozygous mice were generated by mating CCN1*^flox/flox^* C57BL/6J and CAGG-Cre-ERT C57BL/6J mice which donated from Shanghai Jiao Tong University School of Medicine. Sex and age-matched CCN1*^flox/flox^* littermates were used as controls. Mice were maintained under pathogen-free conditions. All animal experiments were performed according to the committee guidelines and approved by the Animal Experiment Center of the Fujian Medical University (SYXK (31) 2016-0006).

### ConA Induced Hepatitis Mice Model

Five- to six-week-old CCN1*^flox/flox^* Cre^+^ and CCN1*^flox/flox^* mice were daily administration of 1 mg/mouse tamoxifen by i.p. for 5 days. Then, mice were injected with 20 mg/kg ConA *via* tail intravenous injection to induce hepatitis mice model. Eight hours later, mice were killed by spinal dislocation after anesthesia, and the liver and serum were collected for follow-up experiments.

### Laboratory Analyses and Assessment

Complete blood cell counts analyses were performed with the ADVIA 2120i Automatic Blood Analyzer (Siemens, Germany). Biochemical examinations were measured with the ADVIA 2400 Biochemical Analyzer (Siemens, Germany). Serum auto-antibodies were quantified by enzyme-linked immunosorbent assays (EUROIMMUNAG, Lubeck, Germany).

### *In Vitro* Cell Experiment

LO2 cells, human liver cell lines (Cell Bank, Chinese Academy of Sciences), were seeded at a density of 1×10^4^/well in 96-well plates and grown in complete DMEM for 24 hours in the presence of serial concentrations of CCN1 (2.5–10 μg/ml, PeproTech, USA). CCN1 small interfering RNA (siRNA) was designed and synthesized at Shanghai GenePharma (Shanghai, China). For the antibody blocking assay, CCN1 protein (5 μg/ml) and anti-α6β1 Ab(20 μg/ml, BioLegend, USA) were premixed for 20 min, and then the mixtures were added to LO2 cells culture system for another 24 h.

### RNA Isolation and Real-Time Quantitative PCR (qPCR)

Total RNA was isolated using TRIzol Reagent (Ambion, Austin, TX, USA) based on phenol/chloroform method. cDNA of mRNA-encoded genes were synthesized using the RevertAid First Strand cDNA Synthesis Kit (ThermoScientific, Lithuania). SYBR Green qPCR were executed using TB Green^®^ Premix Ex Taq™ II (Takara Biotechnology, Dalian, China). Primers were designed using Oligo7 software or obtained from GenBank, and these are listed in **Supplementary Table 1**. Quantitative PCR was performed using a QuantStudio DX and the results were analyzed using QuantStudio Real-Time PCR software (Applied Biosystems Inc., Foster City, CA, USA).

### H&E Staining

Mice liver tissues were removed from sacrificed mice and fixed in 10% phosphate-buffered formalin. Then samples were transferred to ethanol of different concentrations and dimethylbenzene. The sections were embedded with paraffin and stained with HE staining. The slide preparations were made as follow: slides were counterstained with 1% toluidine blue for 5 min, and washed with distilled water and then dehydrated using a graded series of ethanol washes. Haematein solution was added, and the slides were maintained for 5 min following awash with water and hydrochloric acid alcohol. Sections were then stained with eosin solution at 37°C for 5 min, dehydrated with alcohol and immersed in xylene. Finally, the slides were mounted under glass coverslips and analyzed under a light microscope.

### Western Blot Analysis

Denatured protein samples of liver tissue or cell lysates were subjected to 10% SDS-polyacrylamide gel electrophoresis (SDS-PAGE). Proteins were then transferred onto 0.2 μm PVDF membranes (Merck KGaA, Darmstadt, Germany) and blocked with 5% skim milk for at least 1 h at room temperature. Next, the membranes were incubated overnight at 4°C with the first antibody as indicated: 1:1000 CCN1, 1:1000 NF-κB p65, 1:1000 phosphorylated NF-κB p65, 1:1000, Akt, 1:1000 phosphorylated Akt or 1:1000 GAPDH (Cell Signaling Technology, Danvers, MA, USA), 1:500 IKKα/β, 1:500 phosphorylated IKKα/β, 1:500 IκBα, 1:500 phosphorylated IκBα (Beyotime Biotechnology, Shanghai, China). After washing with PBS-Tween (PBST), the membranes were incubated with HRP-conjugated goat anti-rabbit IgG at room temperature for 1h followed by washing with PBST. Immunoblot images were quantified and analyzed using Image Lab software (Bio-Rad Laboratories, CA, USA**).**

### ELISA Detection

Serum of AIH patients, PBC patients, OS patients and HD were collected for the measurement of CCN1 by ELISA (R&D Systems) according to the manufacturer’s recommendations. A standard curve (using a four-parameter logistic curve fit) was performed for each plate and used to calculate the absolute concentrations of CCN1.

### Confocal Laser Scanning Fluorescence Microscopy for NF-κB p65 Nuclear Translocation

LO2 cells grown on glass coverslips were stimulated with 5 μg/ml CCN1 for 60 min. After 4% paraformaldehyde-fixed, 0.5% Triton-X-100-permeablized and PBS/5% BSA-PBS blocked, the cells were stained overnight with anti-NF-κB p65 antibody (Cell Signaling Technology, Danvers, MA, USA), and incubated for 1 additional hour with an Alexa Fluor 594 goat anti-rabbit IgG (H +L) secondary antibody (Invitrogen, Carlsbad, CA, USA). After washing, nuclei were counterstained with DAPI for 20 min. The results were examined using fluorescence confocal microscope. Image acquisition and processing were performed using Zen software (Carl Zeiss Microscopy GmbH, Jena, Germany).

### Immunohistochemical Analysis of CCN1 Expression in Liver Tissue

Liver tissue from AIH patients or ConA-induced hepatitis mice were fixed in 4% paraformaldehyde. Samples were stained with rabbit anti-CCN1 polyclonal antibody (abcam) according to the manufacturer’s instructions. Rabbit IgG were used as negative control. The stained samples were examined by microscopy, and representative sections were photographed. All results of IHC staining score were calculated using Image J software and IHC Profiler plugin (https://sourceforge.net/projects/ihcprofiler). CCN1 expression was classified into 4 levels: negative (IHC score 1), low positive (IHC score 2), positive (IHC score 3) and high positive (IHC score 4).

### Statistical Analysis

Mapping and all statistical analyses were performed using GraphPad Prism 8.0 (GraphPad software, Inc.) or R language (Version R 4.0.3). Data were presented as mean ± SD or n (%). For inter-group comparison, unpaired Student’s t test or Wilcoxon rank-sum test was used dependent on whether data conformed to a normal distribution. Similarly, Analysis of variance (ANOVA) or Mann–Whitney U test was performed for comparisons among multiple groups while correlation analyses was performed using Pearson correlation or Spearman correlation. Categorical data were tested using the chi-square test. To evaluate the diagnostic performance, receiver operating characteristic (ROC) analysis was performed. All statistical tests were two-tailed, and p-value < 0.05 was considered as significant (^∗^ *p* < 0.05, ^∗∗^ *p* < 0.01, ^∗∗∗^ *p* < 0.001).

## Results

### Demographic and Autoantibody Characteristics of AIH Patients, PBC Patients and OS Patients

**Figure 1:**
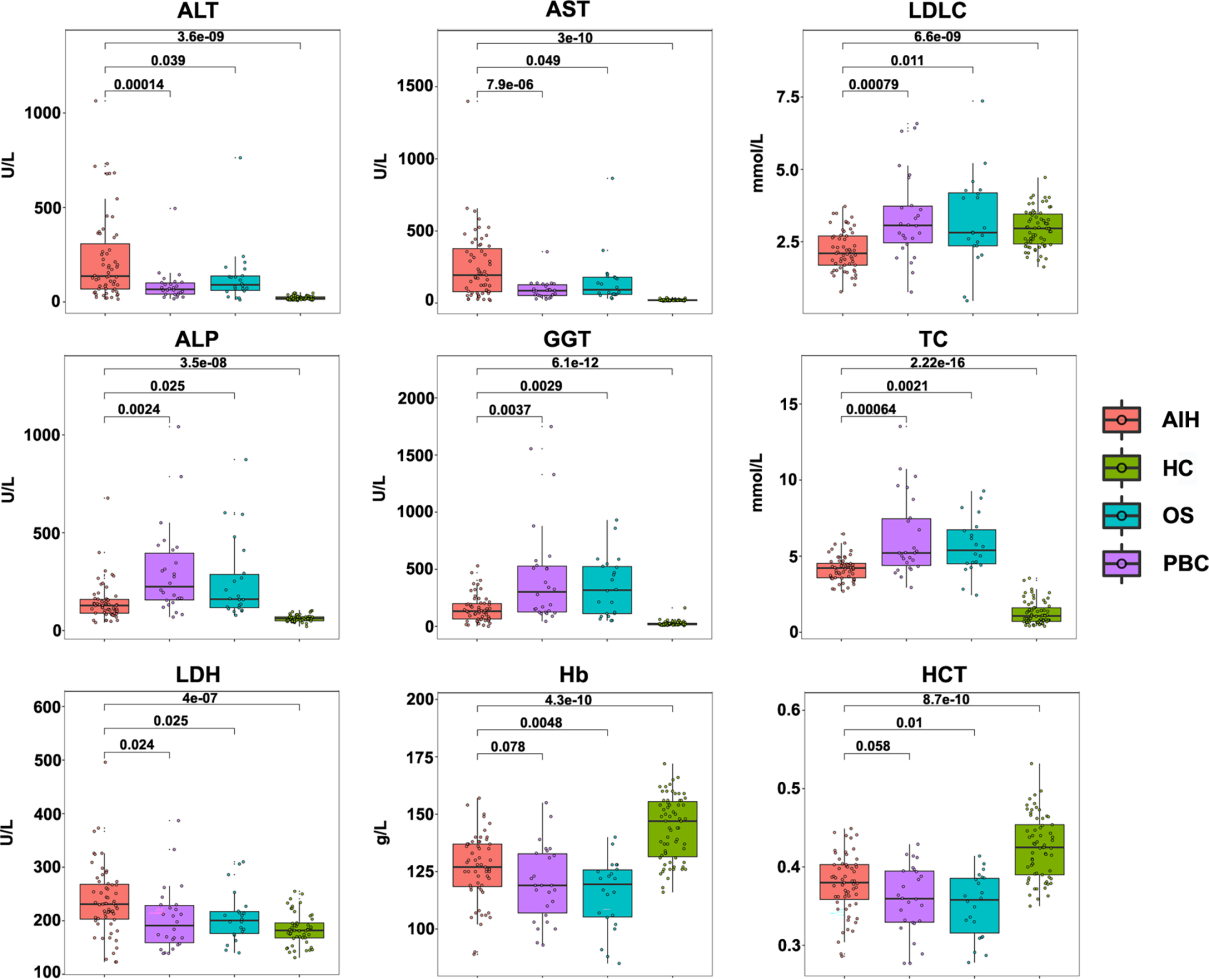
Clinical indicators of AIH patients, PBC patients, OS patients and HC controls. Concentrations of ALT, AST, LDLC, ALP, GGT, TC, LDH, Hb and HCT level in AIH patients (n= 59), PBC patients (n= 26), OS patients (n= 22) and HC controls (n= 68).

The study included data from 59 AIH patients, 26 PBC patients, 22 OS patients and 68 heathy controls. Demographic and clinical data including blood routine examination, liver biochemistry, serum lipids and autoantibodies obtained from medical records were given in **Table 1** and **Figure 1**. Among these clinical indicators, ALT, AST, ALP, GGT, TC and LDH levels were significantly higher, while LDLC, Hb and HCT levels were significantly lower in AIH patients compared to heathy controls.

### The Diagnostic Value of CCN1 in AIH Patients

**Figure 2:**
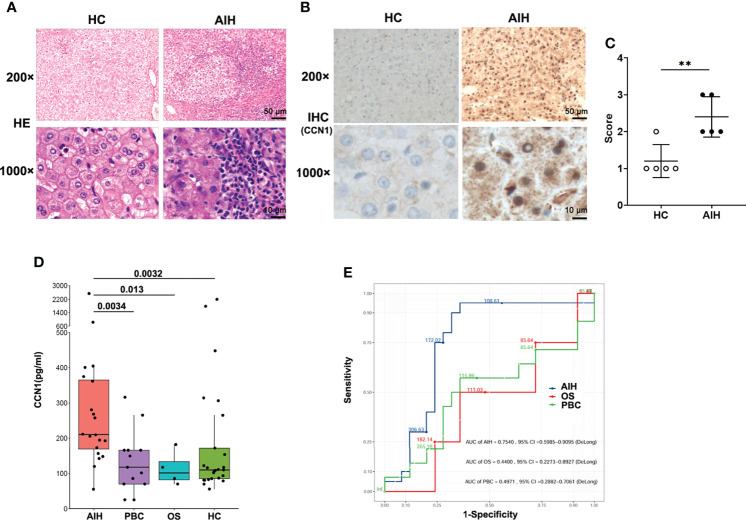
Diagnostic value of CCN1 in AIH patients. **(A)** Representative H&E staining images of liver tissues from healthy control and AIH patients. Original magnification ×200 (upper, scale bar: 50 µm), ×1000 (bottom, scale bar: 10 µm). **(B)** Representative IHC staining images of CCN1 in liver tissues from healthy control and AIH patients. Original magnification ×200 (upper, scale bar: 50 µm), ×1000 (bottom, scale bar: 10 µm). **(C)** Quantification of IHC staining for CCN1 using IHC profiler. The IHC score was expressed as described in methods section. **(D)** CCN1 levels in serum from AIH patients (n= 20), PBC patients (n= 13), OS patients (n= 4) and HC controls (n= 25). **(E)** ROC analysis was performed to evaluate the performance of CCN1 in distinguishing AIH (blue), OS (red) or PBC (green) from HC. **p<0.01.

**Figure 3:**
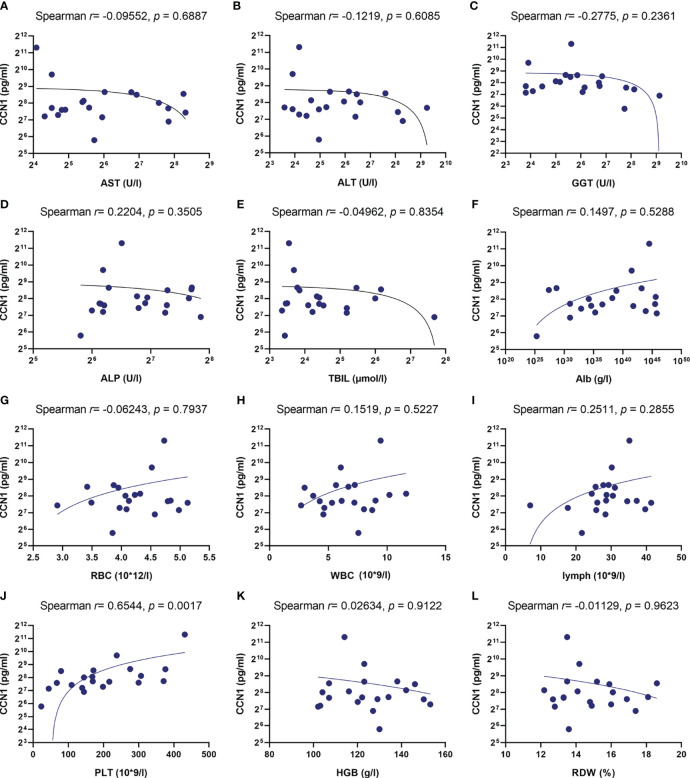
Correlation between CCN1 and liver function and blood routine indexes. **(A-L)** Correlation between CCN1 and AST, ALT, GGT, ALP, TBIL, Alb, RBC, WBC, lymphocyte, PLT, HGB, RDW.

Our previous studies have shown CCN1 was overexpressed in FLS of rheumatoid arthritis (RA) patients. To explore the role of CCN1 in AIH, the protein expression of CCN1 in liver tissue of AIH was examined. As shown in **Figure 2A**, compared to HC, the liver tissue of AIH patients presented with interface hepatitis in pathological section results, which showed that the liver cell necrosis in the portal canal area or adjacent fibrous septa. Also, the portal vein of liver tissues surrounded by dense peripheral infiltrates of inflammatory cells and the focal necrosis as well as bridging necrosis were noticed. Meanwhile, IHC analysis revealed that CCN1 was over-expressed in the cytoplasm of hepatocyte in AIH (**Figures 2B, C**). Moreover, level of CCN1 was significantly higher in the serum of patients with AIH in comparison with HD (**Figure 2D**). ROC curve analysis was performed to evaluate the performance of CCN1 in diagnosis between AIH patients and HC. The area under the curve (AUC) was 0.754, with a 95% confidence interval (CI) ranging from 0.5985 to 0.9095 (**Figure 2E**). Furthermore, we analyzed the correlation between CCN1 and liver function and blood routine indexes. The results showed that CCN1 was positively correlated with PLT (**Figure 3J**). There was no correlation between CCN1 and other indicators (**Figures 3A-L**).

### Knocking Down CCN1 Expression Attenuated Liver Injury and Inflammation in ConA Induced Hepatitis Mice

**Figure 4:**
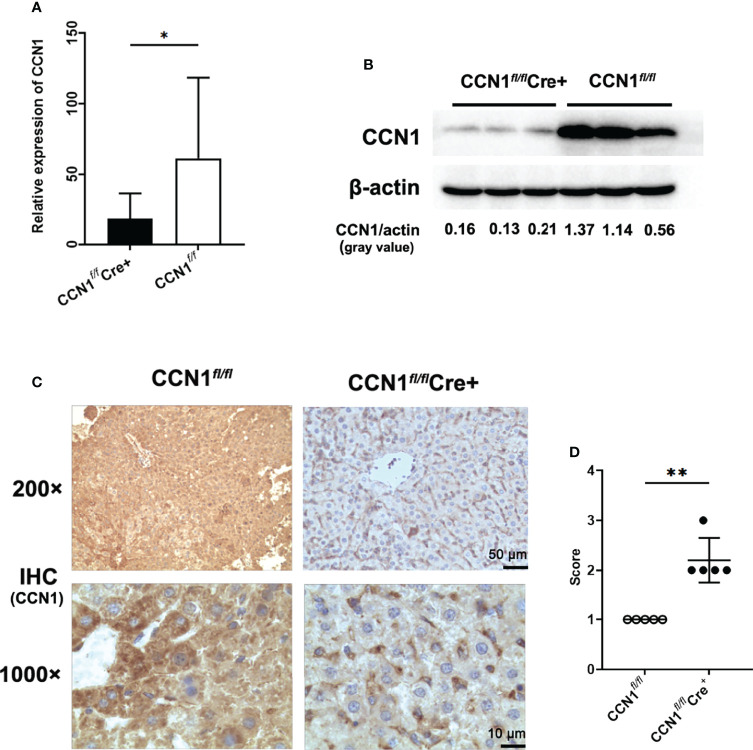
Construction and identification of CCN1 conditional knock out mice. **(A)** mRNA expression of CCN1 in hepatocytes of CCN1*^fl/fl^* Cre^+^ and CCN1*^fl/fl^* mice determined by real-time PCR. **(B)** CCN1 protein level in hepatocytes of CCN1*^fl/fl^* Cre^+^ and CCN1*^fl/fl^* mice detected by Western blot. The relative expression of CCN1 protein was expressed by gray value. **(C)** IHC staining images of CCN1 in liver tissues from CCN1*^fl/fl^* Cre^+^ and CCN1*^fl/fl^* mice. Original magnification ×200 (upper, scale bar: 50 µm), ×1000 (bottom, scale bar: 10 µm). **(D)** Quantification of IHC staining for CCN1 in mice liver using IHC profiler. The IHC score was expressed as described in methods section. Original magnification ×200 (upper), ×1000 (bottom). *p<0.05, **p<0.01.

**Figure 5:**
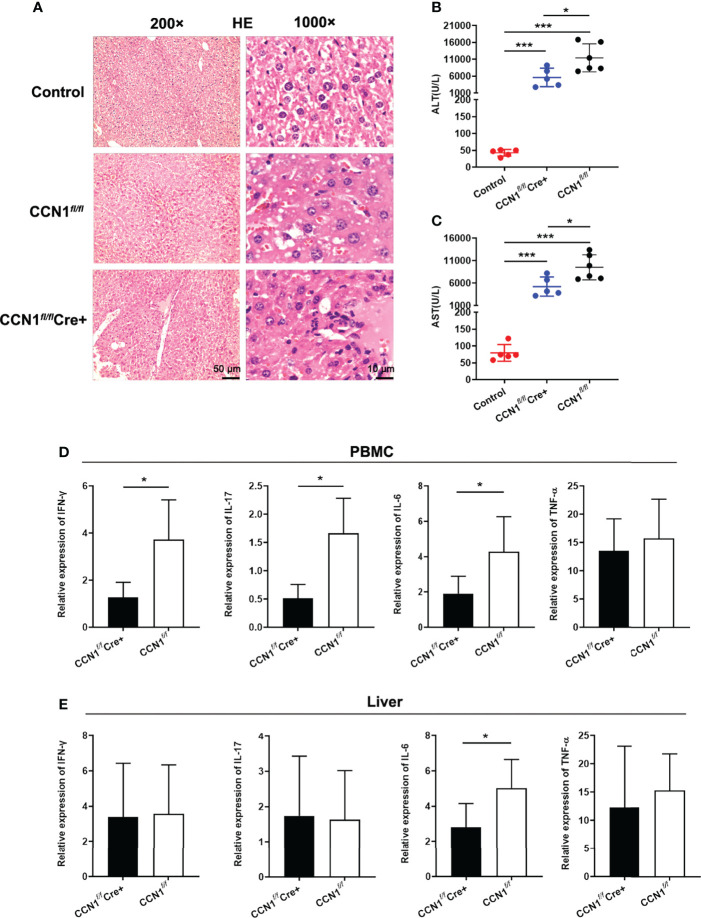
Knocking down CCN1 expression attenuated liver injury and inflammation in ConA induced hepatitis mice. **(A)** Representative H&E staining images of liver tissues from ConA induced hepatitis mice. Original magnification ×200 (left, upper, scale bar: 50 µm), ×1000 (right, scale bar: 10 µm). **(B)** Serum levels of both ALT and AST in CCN1*^fl/fl^* Cre^+^ (blue circle), CCN1*^fl/fl^* (red circle) and control (black circle) mice. **(C)** mRNA expression of inflammatory cytokines IFN-γ, IL-17, IL-6 and TNF-α in PBMC from CCN1*^fl/fl^* Cre^+^ and CCN1*^fl/fl^* mice. **(D)** mRNA expression of IFN-γ, IL-17, IL-6 and TNF-α in PBMC from CCN1*^fl/fl^* Cre^+^ and CCN1*^fl/fl^* mice. **(E)** mRNA expression of IFN-γ, IL-17, IL-6 and TNF-α in liver from CCN1*^fl/fl^* Cre^+^ and CCN1*^fl/fl^* mice. *p<0.05, ***p<0.001.

Considering that CCN1 was overexpressed in hepatocytes and serum of AIH patients, we asked whether CCN1 indeed play roles in inflammation *in vivo*. First, the CCN1 conditional knockout (CCN1*^fl/fl^* Cre^+^) mice were constructed. As shown in **Figures 4A, B**, the mRNA and protein expression of CCN1 reduced in hepatocytes of CCN1*^fl/fl^* Cre^+^ mice. Moreover, the results showed that CCN1 staining was decreased in hepatocytes of CCN1*^fl/fl^* Cre^+^ mice (**Figures 4C, D**). We then used the CCN1 conditional knockout (CCN1*^fl/fl^* Cre^+^) mice to establish ConA induced hepatitis mice model. Histological analysis of the liver tissues was performed by H&E staining to evaluate the pathological changes in ConA induced hepatitis mice. It can be seen that the tissue sections exhibited obvious hepatocyte swelling and inflammatory cell infiltration in ConA induced mice. However, the swelling of hepatocytes and infiltration of inflammatory cells were reduced by CCN1 knocking down (**Figure 5A**). Furthermore, the serum levels of both ALT and AST were much lower in CCN1*^fl/fl^* Cre^+^ mice than that in the CCN1*^fl/fl^* mice (**Figures 5B, C**), indicating that liver injury was alleviated after CCN1 knocking down. The mRNA expression of inflammatory cytokines IFN-γ, IL-17, IL-6 and TNF-α in PBMC and liver were also assessed. It was found that the mRNA expression of IL-6 decreased in both PBMC and liver derived from CCN1*^fl/fl^* Cre^+^ mice compared to that obtained from the control (**Figures 5D, E**). These data indicated that knocking down CCN1 expression could attenuate liver injury and inflammation in ConA induced hepatitis mice.

### CCN1 Induced IL-6 Production in LO2 Cells

**Figure 6:**
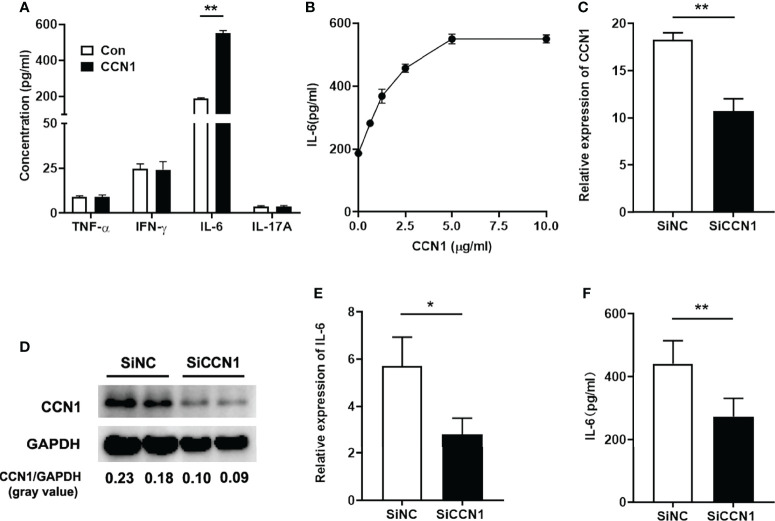
CCN1 induced IL-6 production in LO2 cells. **(A)** IFN-γ, IL-17, IL-6 and TNF-α concentration in supernatant of LO2 cells stimulated by CCN1 (5 μg/ml). **(B)** IL-6 concentration in supernatant of LO2 cells stimulated by CCN1 (2.5, 5, 7.5, 10μg/ml). **(C)** CCN1 mRNA expression in CCN1-knockdown LO2 cells. **(D)** CCN1 protein expression in CCN1-knockdown LO2 cells. The relative expression of CCN1 protein was expressed by gray value. **(E)** IL-6 mRNA expression in LO2 cells treated with SiCCN1 (small interfering RNA against CCN1, black bar) or SiNC (small RNA of negative control, open bar). **(F)** IL-6 level in culture supernatant of LO2 cells treated with SiCCN1 or SiNC. *p<0.05, **p<0.01. All experiments were independently repeated at least three times in triplicate.

As we found that knocking down CCN1 expression could attenuate inflammation by reducing IL-6 expression *in vivo*, we then studied the effect of CCN1 on IL-6 *in vitro* using human liver cell lines LO2 cells. We first tested the potential effect of CCN1 on the protein expression of IFN-γ, IL-17, IL-6 and TNF-α. The results showed that CCN1 stimulation could induce IL-6 production in LO2 cells, but not IFN-γ, IL-17 and TNF-α (**Figure 6A**). Moreover, CCN1 stimulated IL-6 production in LO2 in a dose-dependent manner (**Figure 6B**). To further examine the role of CCN1 in the regulation of IL-6 expression, a specific small interfering RNA (24) was used to knock down CCN1 expression in LO2 cells. As shown in **Figures 6C, D**, the mRNA and protein expression of CCN1 were decreased after CCN1 knockdown. The results showed that IL-6 mRNA expression was remarkably reduced in CCN1-knockdown LO2 cells (**Figure 6E**). The reduction of IL-6 production by LO2 upon CCN1 knockdown was also confirmed by measurement of IL-6 protein levels in culture supernatant (**Figure 6F**).

### CCN1 Induced IL-6 Production in LO2 *via* PI3K/Akt/NF-κB Signaling Pathway

**Figure 7:**
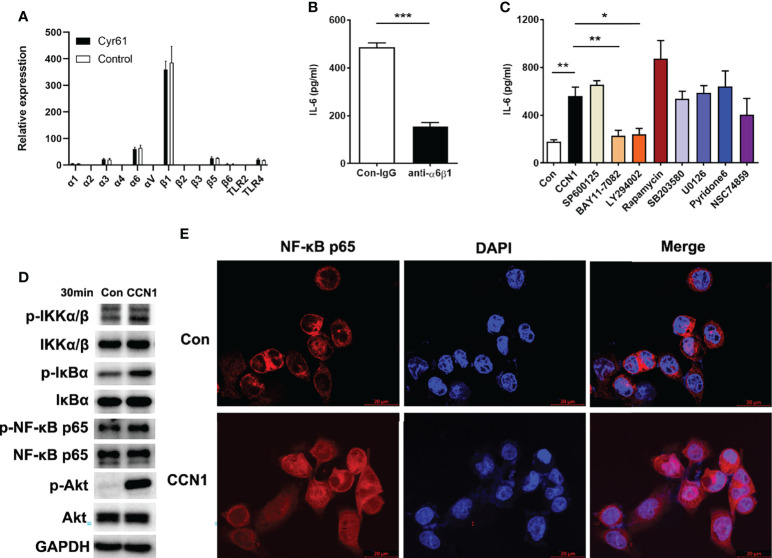
Signaling pathways involved in IL-6 production regulated by CCN1 in LO2 cells. **(A)** Expression of integrins and TLR2/4 in LO2 cells treated with CCN1 or BSA control was determined by real-time PCR. **(B)** Inhibition of CCN1-stimulated IL-6 production by anti-α6β1 mAb. CCN1 protein (5 μg/ml) and anti-α6β1 Ab (20 μg/ml) were premix for 20 min, and then the mixtures were added to LO2 cells culture system for another 24 (h) The culture supernatant was collected for detection of IL-6 protein. **(C)** Inhibitors of signaling pathways on CCN1-induced IL-6 production. LO2 cells were treated with SP600125 (inhibitor of JNK), BAY11-7082 (inhibitor of NF-kB activation), LY294002 (inhibitor of PI3K), SB203580 (inhibitor of p38 MAPK), U0126 (inhibitor of MEK), Pyridone6 (inhibitor of JAK) and NSC74859 (inhibitor of STAT) for 24 (h) The culture supernatant was collected for detection of IL-6. **(D)** Phosphorylation of IKKα/β, IκBα, NF-κB p65 and Akt was detected by Western blot. LO2 cells were stimulated with CCN1 (5 mg/ml) for 30 min. **(E)** Nuclear translocation of NF-κB p65 was monitored by laser confocal fluorescence microscopy. Top panels, Unstimulated LO2. Bottom panels, Stimulated with CCN1 (5 mg/ml) for 60 min. NF-κB was detected by PE–anti-p65 (red). Nuclei were stained with DAPI (blue). Merged picture shows NF-κB translocation into the nucleus. Original magnification ×400. *p<0.05, **p<0.01, ***p<0.001. All experiments were independently repeated at least three times in triplicate.

Several integrins and TLR2/4 have been identified as the cell surface receptors for CCN1 in different cell types (19, 20, 22). Therefore, we first examined the mRNA expression of the integrins and TLR2/4 and found the expression of integrin α6 and β1 was higher in LO2 cells (**Figure 7A**). Moreover, the anti-α6β1 Ab could block CCN1-induced IL-6 production (**Figure 7B**), which suggested that α6β1 was likely the major receptor that mediates the effect of CCN1 on LO2 cells. Further, to probe the downstream signaling pathway(s) of CCN1 in LO2, known inhibitors of several pathways, including SP600125 (inhibitor of JNK) BAY11-7082 (inhibitor of NF-κB activation), LY294002 (inhibitor of PI3K), rapamycin (inhibitor of mTOR), SB203580 (inhibitor of p38 MAPK), U0126 (inhibitor of MEK), Pyridone6 (inhibitor of JAK) and NSC74859 (inhibitor of STAT) were used. The results showed that CCN1 induced IL-6 production in LO2 was markedly decreased in the presence of the NF-κB and PI3K inhibitors (**Figure 7C**). Further analysis showed that CCN1 treatment led to a significant increasement in the phosphorylation level of the IKKα/β, IκBα, NF-κB p65 subunit and Akt in LO2 (**Figure 7D**). Moreover, NF-κB p65 nuclear translocation was enhanced in LO2 cells treated with CCN1 for 30 minutes (**Figure 7E**). Taken together, these results indicated CCN1 induced IL-6 production in LO2 *via* the α6β1/PI3K/Akt/NF-κB signaling pathway.

## Discussion

Our previous studies have demonstrated that CCN1 may act as “proinflammatory factor” to participate in the inflammatory response of RA by inducing IL-6 and IL-8 production (24, 32). Recent studies have shown that CCN1 can induce a ductular reaction during cholestatic diseases, such as PBC (33). Moreover, CCN1 can modulate expansion and a suppressive function of MDSCs in PBC (10). However, the role of CCN1 in AIH has not been fully elucidated. Thus, the study aims to investigate the role of CCN1 in AIH.

Firstly, the clinical general data and test data of 59 patients with AIH, 26 patients with PBC and 22 patients with OS was collected. Statistical analysis showed that ALT, AST, ALP, GGT, TC, LDH, LDLC, Hb and HCT were different among the groups, and the positive rate of anti SMA antibody in AIH was the highest, which was in line with the clinical diagnosis of AIH. On this basis, in order to explore the role of CCN1 in AIH, we collected the liver tissues and serum of AIH patients, and detected the expression of CCN1 in AIH liver tissue and its level in serum. The results showed that CCN1 was highly expressed in AIH patients’ liver tissue, and the level of CCN1 in serum was also significantly higher than that in HD control group. Furthermore, we analyzed the diagnostic efficacy of CCN1 in AIH through ROC curve analysis. The results showed that CCN1 had potential diagnostic value in AIH with the AUC was 0.7540. This part of research shows that CCN1 may play a certain role in AIH, which provides a preliminary basis for our follow-up research.

Then, in order to further study the role of CCN1 in AIH, we constructed CCN1 conditional knockdown mice. The decreased expression of CCN1 in the liver of knockdown mice was verified by qPCR, WB and immunohistochemistry. Using the CCN1 conditional knockout mice, we constructed a ConA induced autoimmune liver mouse model to investigate the role of CCN1 in AIH *in vivo*. The experimental results showed that after knockdown of CCN1, the liver inflammation was reduced, and the levels of ALT and AST in serum were also significantly decreased, which indicated that CCN1 was indeed related to the inflammatory response of liver. How is CCN1 involved in inflammation? Therefore, we also detected the expression of related inflammatory factors in mouse liver and peripheral blood mononuclear cells. The results showed that knockdown of CCN1 expression could reduce the expression of inflammatory factor IFN-γ, IL-17 and IL-6 in peripheral blood, however, only decreased IL-6 expression was observed in liver, suggesting that CCN1 may regulate liver inflammatory response through IL-6 in ConA induced hepatitis mouse model. It has been reported that CCN1 exaggerates ischaemia-reperfusion-induced hepatocyte injury *via* NF-κB/TGF-β1 signalling pathway (34). Combined with our previous studies (24, 25), it is suggested that CCN1 may play roles in different diseases by regulating specific cytokines.

How does CCN1 promote the production of IL-6 in hepatocyte? What is the mechanism? To verify this, we used recombinant CCN1 proteins to stimulate hepatocytes *in vitro* and observed its effect on the production of IL-6. The results showed that CCN1 could stimulate LO2 cells to secrete IL-6 in a concentration- dependent manner. After interfering with the expression of CCN1, the production of IL-6 also decreased, indicating that CCN1 could indeed induce hepatocytes to secrete IL-6. Next, we explored the molecular mechanism of IL-6 production induced by CCN1. We first detected the expression of integrin receptors on the surface of LO2 cells and found that the expression of α6β1 was the highest on LO2 cells. Moreover, α6β1 neutralizing antibody can block the production of IL-6 induced by CCN1, indicating that α6β1 is the receptor of CCN1 on LO2 cells. Further, to investigate the potential mechanism that CCN1 induced IL-6 production, we used known inhibitors of several pathways to study the downstream signaling pathway(s) of CCN1. The results showed that inhibiting NF-κB and PI3K signaling pathways can inhibit CCN1 induced IL-6 production. As Akt is one of the key molecules activated downstream of the PI3K signaling pathway (35, 36), we further examined the phosphorylation of Akt and found the phosphorylated form of Akt was enhanced in response to CCN1 treatment. Moreover, studies have shown that Akt has an important role in the regulation of NF-κB dependent gene transcription. Akt can bind and activate the inhibitor of kappa B kinase-α (IKKα) and thereby promotes the degradation of IκB. which allows NF-κB to translocate to the nucleus and active the transcription of genes (37, 38). Thus, we examined the activation of IKKα/β, IκBα and NF-κB p65. The results showed CCN1 treatment led to a dramatic increase in the phosphorylation level of the IKKα, IκB and NF-κB p65 subunit and enhanced NF-κB p65 nuclear translocation. Notably, our previous and other studies in other cell types also implicated that CCN1 could bind integrin receptors to activate an Akt/NF-κB signaling pathway (24). On the basis of these results, we propose that CCN1 induces IL-6 production in LO2 *via* the α6β1/PI3K/Akt/NF-κB signaling pathway.

Previous studies on CCN1 mainly focused on tumor, tissue damage repair (39, 40), cardiovascular development (41) and so on. In recent years, some studies have focused on the role of CCN1 in inflammation (33, 42). Our previous studies have confirmed that CCN1 plays an important role in the pathogenesis of RA (24–26). The latest study reported from Lester F. Lau also shows that CCN1 is a pattern recognition receiver that opsonizes bacteria for clearance and functions as a damage associated molecular pattern to activate inflammatory responses (20). However, little is known about the study of CCN1 in AIH. As an autoimmune disease, the pathogenesis of AIH is unclear and lack of diagnostic indicators. To our knowledge, the present study preliminarily confirms that CCN1 may be a new diagnostic indicator for AIH, which provides new insight for elucidating the pathogenesis of AIH. However, our study also has some limitations. ConA induced hepatitis mouse model cannot fully reflect the pathogenesis of AIH. The diagnostic value of CCN1 in AIH needs to be confirmed by multi-center studies. The exact mechanism of CCN1 in AIH also needs further research.

**Figure 8:**
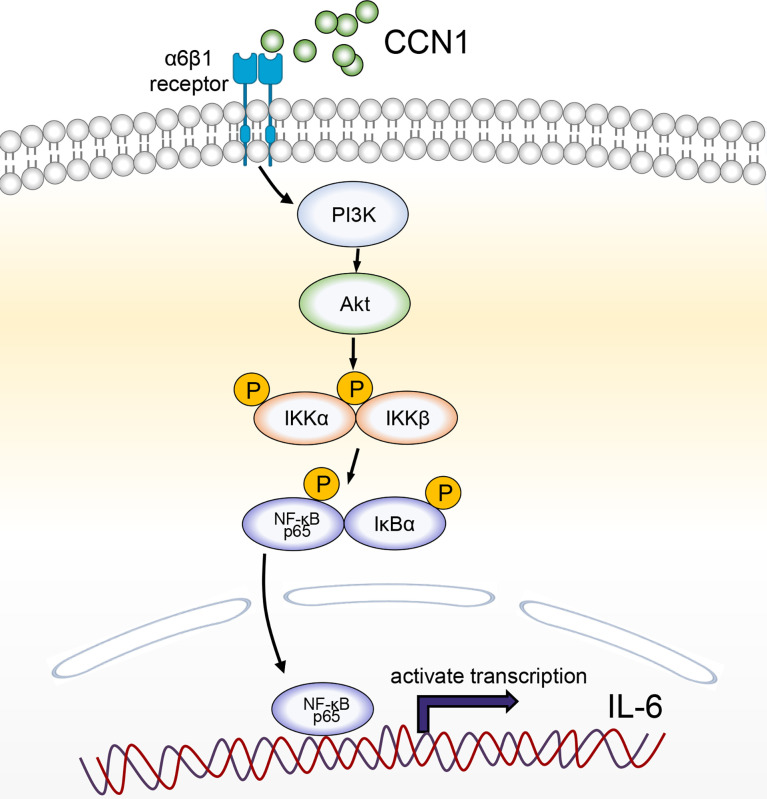
A schematic model for CCN1-stimulated IL-6 production in hepatocytes. CCN1 stimulates IL-6 production *via* α6β1/PI3K/Akt/NF-κB signaling pathway in AIH.

In summary, our study demonstrated that CCN1 could play a certain role in the pathogenesis of AIH by inducing IL-6 production *via* α6β1/PI3K/Akt/NF-κB signaling pathway (**Figure 8**). Targeting CCN1 might be a potential therapeutic strategy for the treatment of AIH.

## Data Availability Statement

The original contributions presented in the study are included in the article/**Supplementary Material**. Further inquiries can be directed to the corresponding authors.

## Ethics Statement

The animal study was reviewed and approved by the Institutional Medical Ethics Review Board of the First Affiliated Hospital of Fujian Medical University, Fuzhou, China (MTCA, ECFAH of FMU [2015]084-1).

## Author Contributions

All authors were involved in drafting the article or revising it critically for important intellectual content, and all authors approved the final version to be published. QO and JL shared the corresponding authorship due to equal essential contributions. They had full access to all the data in the study and takes responsibility for the integrity of the data and the accuracy of the data analysis. RJ and JT developed the concept, conceived the experiments, conducted the majority of the experiments, and completed the manuscript. YH and XZ participated in the experiments and helped with manuscript preparation. ZY, SC, and JX helped with animal housing and collected samples.

## Funding

This work was supported by grant from National Natural Science Foundation of China, China (82072356, 82030063), Program for young and middle-aged backbone of Fujian Provincial Health Commission, China (2019-ZQN-53), Fujian Provincial Health Technology Project, China (2021QNA029).

## Conflict of Interest

The authors declare that the research was conducted in the absence of any commercial or financial relationships that could be construed as a potential conflict of interest.

## Publisher’s Note

All claims expressed in this article are solely those of the authors and do not necessarily represent those of their affiliated organizations, or those of the publisher, the editors and the reviewers. Any product that may be evaluated in this article, or claim that may be made by its manufacturer, is not guaranteed or endorsed by the publisher.

## Abbreviations

AIH, autoimmune hepatitis; CCN1, cellular communication network factor 1; IL-6, Interleukin 6; ROC, receiver operating characteristic; ConA, concanavalin A; FLS, fibroblast-like synoviocytes; PBC, primary biliary cirrhosis; OS, overlap syndrome; ALT, alanine aminotransferase; AST, aspartate aminotransferase; ALP, alkaline phosphatase; LDH, lactate dehydrogenase; TC, total cholesterol; HDLC, high density lipoprotein cholesterol; LDLC, low density lipoprotein cholesterol; RA, rheumatoid arthritis.
