# Supplementary material for: CCN1 Promotes Inflammation by Inducing IL-6 Production via α6β1/PI3K/Akt/NF-κB Pathway in Autoimmune Hepatitis
Source: Front Immunol. 2022 Apr 25;13:810671. doi: 10.3389/fimmu.2022.810671 (PMC9084230; doi:10.3389/fimmu.2022.810671)
Supplement: Supplementary file 1 [file Table_1.docx]

**Supplementary Table 1**

Primers used for qPCR or plasmids cloning.

| Species | Gene Name | Sequence (5′-3′) |
| --- | --- | --- |
| Homo  sapiens | CCN1 | Fw: GGTCAAAGTTACCGGGCAGT |
|  |  | Rv: GGAGGCATCGAATCCCAGC |
|  | IL-6 | Fw: CCGGGAACGAAAGAGAAGCT |
|  |  | Rv: GCGCTTGTGGAGAAGGAGTT |
|  | Integrin α1 | Fw: ACAAGGGAGACAGTGCGTACAA |
|  |  | Rv: TTGATTGGATTCGCAGCTATCTT |
|  | Integrin α2 | Fw: GAGAACCGAATGGGAGATGTG |
|  |  | Rv: TTTGAGATGCTTGCTGAATTTTG |
|  | Integrin α3 | Fw: ATCATCCTCCTCTTGTGGAAGTG |
|  |  | Rv: AGCCTTCTGCCTCTTAGCTTCAT |
|  | Integrin α4 | Fw: CGAGACATCCTTACCCCCATT |
|  |  | Rv: AAATTCCTCAGTGTTTCGTTTGG |
|  | Integrin α6 | Fw: CACTAACAGAGTGGCCGTCCTA |
|  |  | Rv: CCCCAATGGCATAGTCTTGTG |
|  | Integrin αv | Fw: TGTTACCCTTTGGCCTTCTTTCT |
|  |  | Rv: GACAAGGGAGACGGGAACCT |
|  | Integrin β1 | Fw: AACGAGGTCATGGTTCATGTTG |
|  |  | Rv: CAAGGCCAATAAGAACAATTCCA |
|  | Integrin β2 | Fw: CACACAAACTTTCCGAGAGCAA |
|  |  | Rv: TTTCTCATACGTTTTCACCATCTTCT |
|  | Integrin β3 | Fw: CACCATCCACGACCGAAAAG |
|  |  | Rv: GGTACGTGATATTGGTGAAGGTAGAC |
|  | Integrin β5 | Fw: GGATTTGGGTCTTTTGTTGATAAGG |
|  |  | Rv: AATGCACGGATTGGTCTGGTA |
|  | Integrin β6 | Fw: GTGTCCTCTTCCCACACCAAA |
|  |  | Rv: CCCCACTGGCTTTATGATGAG |
|  | TLR2 | Fw: GAACTTATCCAGCACACGAA |
|  |  | Rv: AGTTGCTCCTTAGAAAACGTA |
|  | TLR4 | Fw: TGACCATTGAAGAATTCCGAT |
|  |  | Rv: ATTCTAAATGTTGCCATCCG |
|  | GAPDH | Fw: GGAGCGAGATCCCTCCAAAAT |
|  |  | Rv: GGCTGTTGTCATACTTCTCATGG |
| Mus | CCN1 | Fw: TAAGGTCTGCGCTAAACAACTC |
| musculus |  | Rv: CGTCCAGGGAGTCCTTAATGC |
|  | IL-17A | Fw: TTTAACTCCCTTGGCGCAAAA  Rv: CTTTCCCTCCGCATTGACAC |
|  | IFN-γ | Fw: GCTCTGAGACAATGAACGCTA |
|  |  | Rv: TTTTCTTCCACATCTATGCCAC |
|  | IL-6 | Fw: CCTCACACGAGGCACAAGTG |
|  |  | Rv: CTCTCCCTGGACTCATGTTTGC |
|  | TNF-α | Fw: GCCTGTACCTCATCTACTCC |
|  |  | Rv: CAGATAGATGGGCTCATACCAG |
|  | β-actin | Fw: TGTCCACCTTCCAGCAGATGT |
|  |  | Rv: AGCTCAGTA ACAGTCCGCCTAG |
|  | SiCCN1 | sense: GGAGUUGACGAGAAACAAUTT |
|  |  | antisense: AUUGUUUCUCGUCAACUCCTT |

Abbreviations: TLR: Toll-like receptor; Fw, Forward primer; Rv: Reverse primer; siRNA, short interfering RNA; NC, normal control.
